# Generic Emergence of Modularity in Spatial Networks

**DOI:** 10.1038/s41598-020-65669-8

**Published:** 2020-05-26

**Authors:** Luis J. Gilarranz

**Affiliations:** Department of Aquatic Ecology, Eawag (Swiss Federal Institute of Aquatic Science and Technology) Überlandstrasse 133, 8600 Dübendorf, ZH Switzerland

**Keywords:** Ecological networks, Theoretical ecology

## Abstract

Landscape’s spatial structure has vast implications for the dynamics and distribution of species populations and ecological communities. However, the characterization of the structure of spatial networks has not received nearly as much attention as networks of species interactions counterparts. Recent experiments show the dynamical implications of modularity to buffer perturbations, and theory shows that several other processes might be impacted if spatial networks were modular, from disease transmission to gene flow. Yet the question is, are spatial networks actually modular? Even though some case studies have found modular structures, we lack a general answer to that question. Here, I show that modularity is a naturally emergent property of spatial networks. This finding is further reinforced by analyzing real patchy habitats. Furthermore, I show that there is no need for any other biological process other than dispersal in order to generate a significantly modular spatial network. Modularity is explained by the spatial heterogeneity in the density of habitat fragments. The fact that spatial networks are intrinsically modular might have direct consequences for population and evolutionary dynamics. Modules define the spatial limits of populations and the role each habitat fragment plays in ecological dynamics; they become the relevant scale at which a multitude of processes occur.

## Introduction

For quite some time, ecologists neglected the role of spatial dynamics^1^. Yet, all ecological and evolutionary processes occur within the landscape^2^. The spatial dimension can modulate species survival and the outcome of interactions^3^. Consequently, we cannot fully understand ecological communities out of the spatial context in which they occur.

Following the parallel development of complex networks in ecology, biotic and spatial networks came to respectively describe the way in which species interact, and how habitat fragments relate to one another. By analyzing biotic networks, ecologists have discovered several universal patterns. Both food webs and mutualistic networks are modular^4,5^, and follow highly heterogeneous degree distributions^6,7^, while spatial networks follow a Poisson distribution^8^ (Supplementary Fig. 1). All these patterns have been found in empirical networks, and there is solid theoretical work describing their dynamical consequences^9–14^. However, only the dynamical effects of modular structures have been tested experimentally^15^. That experiment was not conducted on food webs or mutualistic networks—due to the difficulty of experimentally manipulating species interactions in large communities^16^—but by using spatial networks as a surrogate. If we are meant to translate those findings for the understanding, management, and design of spatial networks, one question remains. Are spatial networks even modular?

Spatial networks are sets of nodes (i.e., habitat fragments or patches) whose coordinates are known and where links represent dispersal routes between habitat fragments^17^. They are the template over which metapopulation and metacommunity dynamics operate. They have profound implications for the persistence of populations^18,19^, for the spatial distribution of species, interactions, and the organization of biological communities^20–22^. If those spatial networks had a modular structure—where habitat fragments would tend to be organized in modules where nodes inside one module interact more frequently with nodes from the same module than from nodes from a different module—theory shows that the spread of diseases will be slowed^11^, perturbations buffered^15^, and large-scale outbreaks explained^23^. Also, if spatial networks were modular, then the relevant scale for a plethora of ecological processes will be the module.

In a few study organisms at certain locations, significant modularity has been found in their respective spatial networks^24,25^. However, there is a lack of understanding of the processes that lead to that structure and whether it is a generalized phenomenon or if, on the contrary, it is anecdotal. In contrast with networks of species interactions, whose patterns originate through biological processes mediated by coevolution, specialization, or body size^26–28^, the structure of spatial networks is just the result of the dispersal between habitat fragments, whose location is given.

I aim to shed light into these questions by creating generic artificial landscapes and analyzing real ones. Studying the interplay between dispersal and landscape properties allow me to understand the emergence of modularity in the landscape.

## Results

**Figure 1:**
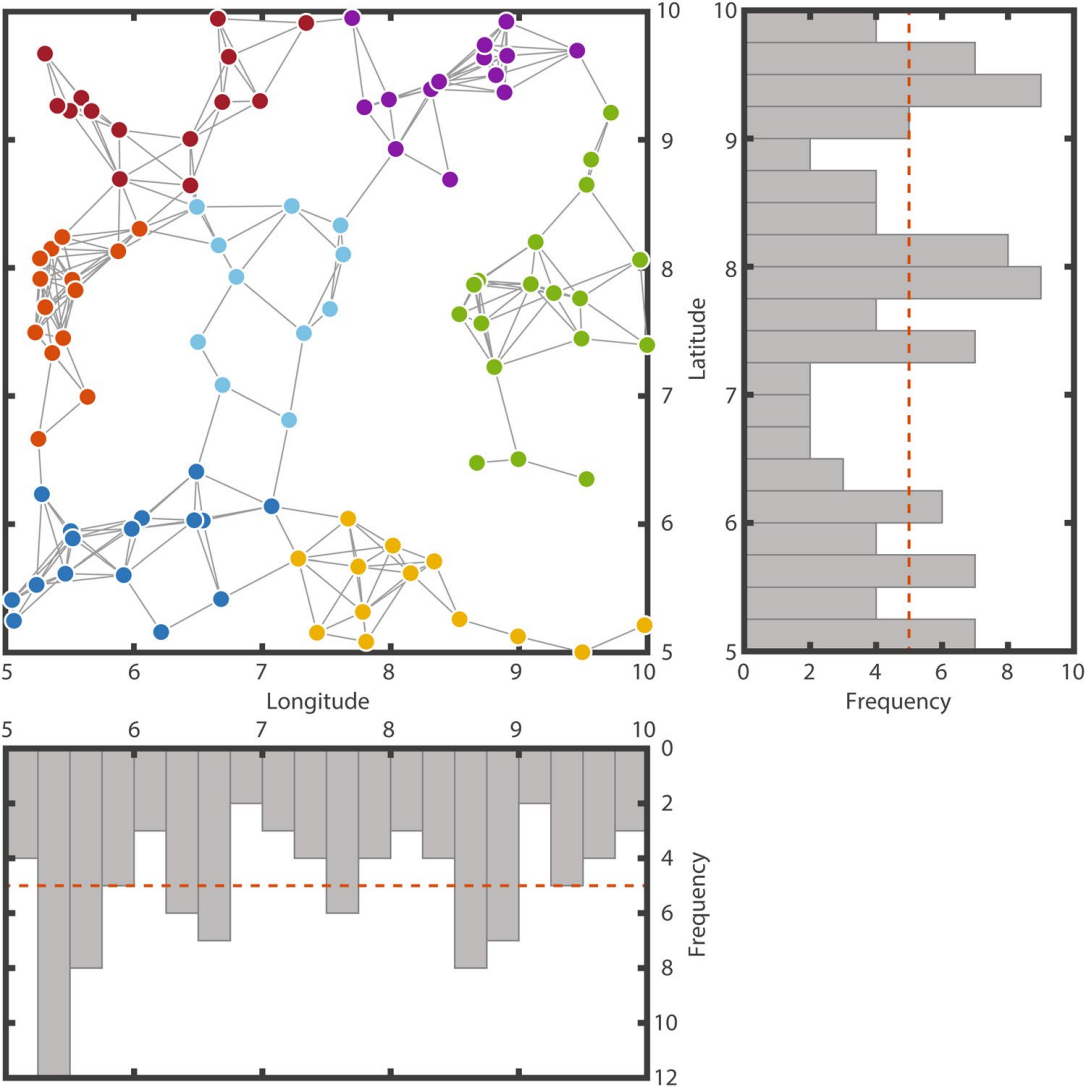
Modular network structure and spatial distribution of coordinates. The main panel shows 100 nodes with their respective coordinates linked by the percolation graph. This is the spatial network created using the percolation distance as a threshold distance. Node colors represent the module partition that maximizes modularity. Both histograms show the density of nodes across longitude and latitude. The red dotted line represents the theoretical uniform distribution from which the coordinates are drawn.

Here, I show that modularity is a naturally emergent property of spatial networks. Even in the simplest scenario, where coordinates of habitat fragments are drawn from a uniform distribution, non-random modular patterns deterministically emerge regardless of the distance at which a species can disperse (Fig. 1). While networks of species interactions need a feedback mechanism for modular structures to evolve^29^, spatial networks are modular even when dispersal is the only generative process. These findings are further corroborated by analyzing real patchy landscapes (Supplementary Figs. 3 and 4).

**Figure 2:**
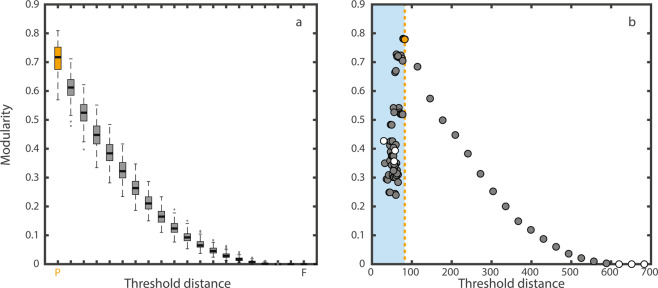
Modularity as a function of the threshold distance. (**a**) The plot shows the results of over 100 random landscapes (each with 100 habitat fragments) with coordinates drawn from uniform distributions. In each case, the network with the highest modularity corresponds to the percolation distance (orange P). Since each landscape has a different percolation distance, the boxplot shows a relative increase of distance from the percolation distance P to the distance which generates a fully connected matrix F and 20 other distances equally spaced in between. Modularity is significant throughout nearly the entire range of threshold distances. The pervasiveness of the pattern shows that it is scale-invariant. Increasing threshold distance is equivalent to reducing the scale at which we look at the landscape. However, threshold distances close to the distance in which the resulting graph is a fully connected network, result in a non-significant modular pattern. (**b)** The plot represents the random landscape depicted in Fig. 1. For distances shorter than the percolation distance (blue-shaded area) the different components also tend to have a modular structure. Modularity increases as the size of the components increase until it peaks at around the percolation distance (orange dotted line). Grey circles represent significant values of modularity, while white circles represent non-significant values. (A zoomed-in version of the distances shorter than the percolation distance, and the size of each individual component can be found at Supplementary Fig. 2).

As the threshold dispersal distance—the distance over which we assume no dispersal—changes, the properties of the spatial network change with it^30^. The percolation graph—being the spatial network created with the percolation distance as a threshold^17,31^—returns the highest level of modularity (Fig. 2). As the threshold distance increases, modularity decreases exponentially^32^. An increase in dispersal distance blurs the limits between modules, increasing movement across modules. This result is coherent with field observations showing that the shorter the dispersal distance, the larger the spatial genetic heterogeneity found in the landscape^33^. These results also hold true when the coordinates are drawn from a normal—instead of uniform—distribution (Supplementary Fig. 5), and for real empirical landscapes (Supplementary Fig. 7).

Modularity remains significantly larger than the random expectations almost until the graph is fully connected. To test the observed network against an adequate null model (see Methods), is not only the number of nodes and links preserved in the randomized networks but also the degree of each node. These randomizations do not account for distance when rewiring the network. Therefore, randomizing the spatial networks in this way is equivalent to testing the effect of the spatial restrictions (coordinates and threshold distance) on the topology of the network. The results presented here show that these restrictions are the ones responsible for the modular pattern.

**Figure 3:**
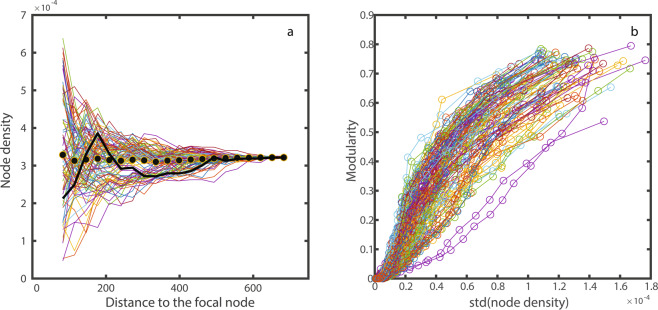
Explaining the emergence of modularity. (**a**) For a certain random landscape (the one shown in Fig. 1), average node densities as a function of the distance to a focal node (equivalent to threshold distance in Fig. 2). Each solid line represents the density of nodes (nodes/Km^2^) around a specific node in the landscape. Black dots represent the average density experience across all nodes. The thick black line highlights a particular node, one can see how the density can be lower or larger than the landscape average as a function of the distance to that focal node. Only for very large distances, the density converges to the average. To calculate node density, the area considered around the focal node was only the area that fell within the latitude and longitude limits of the landscape. Variance in node density is higher, the closer we look around a specific point in the landscape. (**b)** The modularity of the spatial network can be explained by the standard deviation of the node density around the nodes in the landscape. This panel shows a hundred different landscapes (each with 100 habitat fragments), where each line represents a different random landscape.

Therefore, understanding the distribution of node coordinates is key to explaining why modularity emerges in spatial networks. A single realization of a uniform distribution inevitably contains random deviations from that distribution. Those deviations generate small density fluctuations in space (histograms in Fig. 1). We observe how, for an individual realization of the distributions of points in space, the density of points deviates locally from the average. These random fluctuations are created because even though the probability distribution of points in space is homogeneous, this is only true when the number of points tends to infinity. When the number of nodes in the network is finite, these fluctuations local aggregations of nodes followed by local empty spaces (Fig. 3a). These spatial heterogeneity in return create fluctuations in the establishment of dispersal routes, and are ultimately responsible for the topological modules in the spatial networks^34^. The aggregations turn to be the modules while the empty spaces are the limits between modules.

As Fig. 3a shows, the density of nodes (number of nodes/Km^2^) within a radius around a focal node deviates from the global average density. As the radius considered increases, the observed density around a specific focal node can shift even between larger and lower values compared with the average global density. For a certain threshold distance *t*, we can quantify these local fluctuations in node density as the standard deviation ${\sigma }_{t}$. Given the formula for the standard deviation, $\sigma (t)=\,\sqrt{\frac{1}{N-1}\mathop{\sum }\limits_{i=1}^{N}{({x}_{i}-\bar{x})}^{2}}$, where *N* is the number of habitat fragments in the landscape, and *x*_*i*_ is the density of nodes around an habitat fragment *i* considering a radius equal to the threshold distance considered. Supplementary Fig. 6 quantifies how the standard deviation in node density decreases with the threshold distance. Local node density only converges to the global average once the radius is almost as big as the entire landscape. This property is ultimately responsible for the apparent relationship between modularity and threshold distance. Figure 3b shows the positive relationship between modularity and the standard deviation of node density. Even though the exact shape of that relationship is landscape-dependent, the spearman’s rank correlation between modularity and the standard deviation of node density is 1 for every individual landscape, revealing a strictly monotonous relationship.

**Figure 4:**
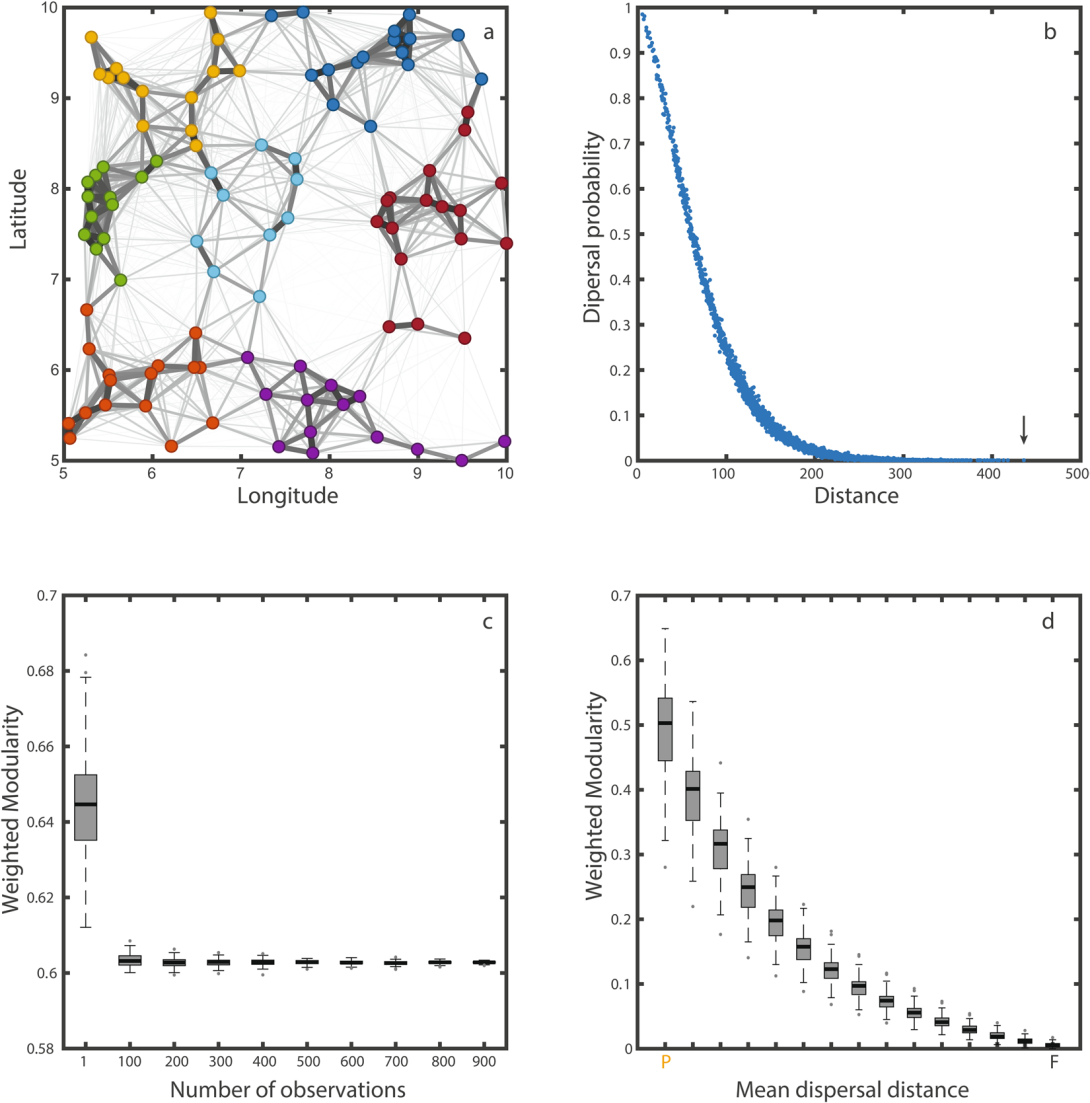
Dispersal kernel instead of dispersal threshold. (**a**) nodes with their respective coordinates linked by a probabilistic dispersal network. Link width is proportional to the frequency at which two nodes are connected when drawing random dispersal events following a Weibull distribution (methods). Node colors represent the module partition that maximize modularity in weighted networks. (**b)** Posterior dispersal kernel obtained from panel a. It shows the dispersal probability as a function of distance between nodes. The arrow points the maximum dispersal distance recorded (**c)** Convergence of the value of modularity as a function of the number of observations. Each observation is a binary network where a link is established as a function of a probability dispersal kernel. The number of observations represent how many of these networks we add together to create the weighted network over which we calculate modularity. The distributions correspond to combinations of 100 weighted networks. (**d)** Analogous to Fig. 2a. It shows how modularity decreases as a function of mean of the Weibull distribution used to determine whether a link is established for 100 random landscapes. For comparison purposes, each of the individual binary networks have the same connectance that those in Fig. 2a and the mean dispersal distances coincides with the threshold distance.

These results so far, show how the landscape is perceived at the limit, when dispersal corresponds to the maximum possible dispersal distance. These binary spatial networks represent the scale of the landscape at equilibrium, over long enough time, and for a large enough number of individuals. For shorter time scales or a fewer number of individuals, a probabilistic approach is necessary. Dispersal kernels represent the probability of dispersal of a species as a function of the distance^35^. Ecological and evolutionary pressures affect not only the maximum dispersal distance but also dispersal probability at intermediate distances^36^. Under this framework, we can generate probabilistic spatial networks (Fig. 4a), where link weight represents the probability or frequency of dispersal events between two nodes (see methods). These networks represent what we would observe in field studies^37^. Also, in agreement with field observations, the observed dispersal probability decays with distance^38^, as shown in Fig. 4b.

The modularity of the probabilistic dispersal network quickly converges as the number of recorded dispersal events increases (Fig. 4c). This implies that not many observations are needed to obtain a good estimate of modularity. Figure 4d shows that regardless of how the spatial network is obtained, the findings reported here hold, as the weighted modularity also decreases with average dispersal distance.

Moreover, the emergence of modularity is not unique to the theoretical coordinates explored here. The same results are found when dealing with coordinates extracted from real habitat fragments like mountain tops in the Alps or lakes in Siberia (Supplementary Figs. 3 and 4). These examples add to the case studies previously reported in the literature^24,25^, reinforcing the evidence that patchy and fragmented landscapes are inherently modular without the need for a process other than dispersal.

The fact that spatial networks are modular implies that we can partition the landscape in distinct modules. The partition of the network in modules tells us which habitat fragments group together, revealing the spatial limits of species populations. The level of modularity tells us how isolated those populations are. Revealing the subdivision of the landscape into modules may also help explain patterns at the node level as not all have the same role in the network. Different habitat fragments behave differently regarding the presence of certain species or the organization of the local biological communities^39^. Of particular interest are the fragments which bridge between modules—these nodes have links to more than one module. They are sentinel nodes, mathematically defined as nodes whose participation coefficient is different from zero. In the event of a perturbation spreading between modules (i.e., a disease, or an invasive species), these sentinel nodes will be affected before the rest of the module is^15^. Monitoring these nodes will provide an early warning as they indicate that a perturbation has jumped from one module to another. In Fig. 1, the sentinel nodes are only 22% of the total number of nodes. The larger the modularity, the smaller the fraction of sentinel nodes in a network. If monitoring all the sentinel nodes is not feasible, the participation coefficient value provides a ranking informing on which nodes should be preferentially surveilled.

## Discussion

Here I’ve shown that modularity is a general property of spatial networks. This pattern has dynamical consequences at the landscape level, from slowing down the spread of diseases^11^, to diminishing expected population abundances^15^. But more importantly, a modular network implies a mesoscale in the landscape. A plethora of ecological and evolutionary processes can be better understood when considering the modules in which the landscape is subdivided. The aforementioned disease and metapopulation dynamics can only be understood in the context of modularity hindering dispersal between modules while favoring dispersal within modules. The same phenomenon leads to reproductive isolation between local populations inhabiting different modules.

From an evolutionary perspective, the maximum dispersal distance is a key trait over which species may encounter selection pressures^40^. For dispersal distances where we find a pronounced modular pattern, the frequency of encounters between populations of the same module will be higher than across modules. This might be enough to explain genetic differences between populations, affecting evolution, and eventually speciation. In this context, modules in spatial networks have been suggested to be a natural definition of populations as they may constitute evolutionary units^24,41^.

Spatial networks act as an invisible road map that links habitat fragments together. Revealing their universal structure is a step towards a better understanding of spatial dynamics and the distribution of species and communities across space. To operationalize these findings, we should better understand the interplay between spatial structure and the parameters of dynamical models. The same output could be the result of a different spatial structure or of a different dynamical properties of the population utilizing the space^19,42^. This is why we should improve our understanding of dispersal, not just for individual species, but for all the species involved in a community. We need to regard dispersal kernels as key traits. At the same time, we need to expand the findings presented in this paper to entire communities, profiting from multilayer network approaches where each layer will represent a species in that community. This will help us understand the spatial scale of species’ interactions and improve our predictions on metacommunity dynamics.

Beyond ecology, spatial networks—understood as mathematical objects—are useful descriptors of a plethora of systems. Therefore, the finding that random spatial networks are modular by construction transcends the realm of ecology, with implications spanning from urban planning to logistics and economics^43–45^.

## Materials and Methods

### Creating spatial networks

The main challenge is gathering knowledge of the actual dispersal routes. In the simplest case, links are established as a function of the distance between nodes^30,46–48^. Other approaches have used genetic similarity^24,25,41,49^, capture-recapture^25^, or seed traps and genetic analysis^50^ to establish links between habitat fragments.

Here I use two approaches to create spatial networks. First, the limit case in which links are established through a distance threshold. If the distance between two habitat fragments is shorter than the threshold dispersal distance, a link is established^30,46–48^. Spatial networks are a binary unipartite undirected adjacency matrix where 1 means that there is a dispersal route between two nodes because the distance between those two nodes is smaller than the distance threshold.

The second approach creates probabilistic dispersal networks. Evidence shows that even if there is a characteristic maximum distance over which a certain species cannot disperse, dispersal probability changes as a function of the distance between nodes. Moreover, evidence suggests that such dispersal kernels (probability function) are heterogeneous^35^. Following this evidence, I create probabilistic dispersal networks in the following way:

First, for every possible link, I establish whether there is a dispersal link or not as a function of a Weibull distribution with scale parameter *a* = *t*, and shape parameter *b* = 1.5^51^. For comparison purposes, the scale parameter corresponds to the distance threshold *t* used for the non-probabilistic spatial networks. I draw pairs of nodes at random and establish whether there is a link or not between them as a function of the dispersal kernel until two conditions are met: the network is connected in a single giant component, and the number of links is the same as in the non-probabilistic versions of the spatial networks for *a* = *t*. This results in a binary network with the same connectance as the binary networks obtained using a distance threshold.

Second, the same procedure is repeated 100 times resulting in 100 binary networks. Each of these networks is an observation of a probabilistic dispersal network. In order to obtain the weighted dispersal network, I add all the adjacency matrices of these binary networks, resulting in a network *W* where elements *w*_*i,j*_ represent the frequency at which two nodes are connected when drawing random dispersal events following the aforementioned dispersal kernel.

### Calculating modularity

To calculate modularity, one has to find a network partition into modules that maximize the value of M (Eq. 2)^52,53^. This partition is the one that groups the nodes in a way in which the fraction of links between modules is minimal when compared with the fraction of links inside modules:

1 $$M\equiv \mathop{\sum }\limits_{s=1}^{{N}_{M}}\left[\frac{{l}_{s}}{L}-\,{\left(\frac{{d}_{s}}{2L}\right)}^{2}\right]$$

*N*_*M*_ is the number of modules, *L* is the number of links in the entire component, ${l}_{s}$ is the number of links between nodes that belong to module *s*, and *d*_*s*_ is the sum of the degrees of the nodes that belong to module $s$. The extension to weighted networks takes the weighted network instead of the binary adjacency matrix^54^. To obtain the optimal partition, several algorithms have been proposed over the years, being the one proposed in ref. ^44^, based on simulated annealing^55^, the best performing one for unipartite, undirected networks. Due to the resolution limit in community detection^56^, only components with at least 10 nodes were analyzed (Supplementary Fig. 2).

To assess whether the value of modularity found is different from what would be expected by chance, a comparison with an adequate null model is necessary^34^. To fall on the conservative side, I kept as many parameters fixed as possible by using the swap algorithm^57^, so that each network is compared with 100 other randomized versions of it with the same number of nodes, the same number of links, and same degree sequence. A value of modularity is considered to be significantly different from these random expectations when the z-score falls off the ±1.96 values that correspond to the 95% confidence intervals.

Note that the goal of this methodology is solely to look for patterns that are due to the spatial distribution of nodes. This would detect the baseline modularity of spatial configurations, a null model relevant when trying to detect patterns that are not due to mere physical proximity^58,59^.

Sentinel nodes are defined as nodes whose participation coefficient is different from zero. These are nodes that have links with nodes from more than one module. I define the participation coefficient following ref. ^53^. The participation coefficient ${P}_{i}$ is defined as

2 $${P}_{i}=1-\mathop{\sum }\limits_{s=1}^{{N}_{M}}{\left(\frac{{k}_{is}}{{k}_{i}}\right)}^{2}$$

where ${k}_{is}$ is the number of links of node *i* to nodes in the module to which *i* belongs, and ${k}_{i}$ is the total number of links of node *i*.

### Calculating distance between coordinate pairs

To calculate the distance between each pair of coordinates, I calculate the great circle. A great circle is the shortest path between two points along the surface of a sphere. Even though Euclidean distances and great circle distances are the same for nearby locations, using great circles allows us to generalize the method.

### Calculating coordinates of lakes in Yamal, Siberia

To analyze the modularity of the spatial network of the lakes in Yamal, Siberia (Russia), I obtained the centroid of each lake from the available satellite data as a reference to represent the lake on the figure^60^. The distance between every lake was calculated based on the shortest distances between their shores (Supplementary Fig. 4). The large number of habitat fragments in both the Alps and Siberian lakes allow us to show that the distribution of coordinates in both cases follow a uniformly random distribution, providing support for the use of this probability distribution when drawing the coordinates of the synthetic landscapes.

## Supplementary information

Supplementary Information.

## Data Availability

The code written for this manuscript will be available upon request.
