# Supplementary material for: Generic Emergence of Modularity in Spatial Networks
Source: Sci Rep. 2020 May 26;10:8708. doi: 10.1038/s41598-020-65669-8 (PMC7250921; doi:10.1038/s41598-020-65669-8)

Supplementary Information for

Generic emergence of modularity in spatial networks

Luis J. Gilarranz
Email: luis.gilarranz@eawag.ch

Supplementary Figure 1 | Threshold distance changes the properties of the spatial network. Although the degree distribution of all random geometric graphs follows a Poisson distribution, the average degree and the degree range (maximum-minimum degree) changes greatly with the threshold distance. As the average degree increases, modularity decreases. In this figure, I explore threshold distances from the threshold distance to the distance at which the spatial network is fully connected. The average degree increases with distance while the degree range finds a maximum value around 300m.

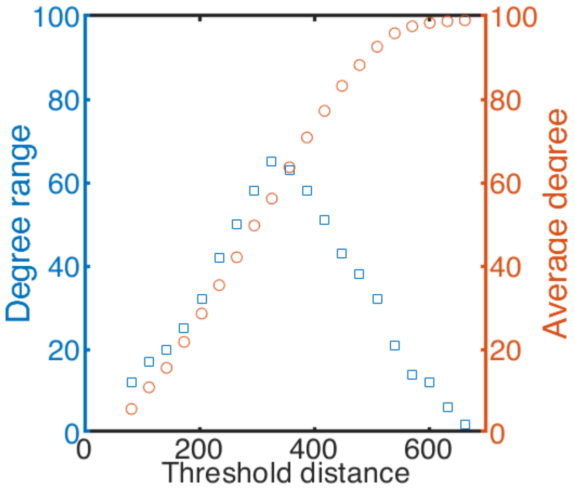

Supplementary Figure 2 | Exploring the spatial network for threshold distances shorter than the percolation distance. Size (**a**) and modularity values (**b**) of each component of the spatial network shown in figure 2b in the main text. White circles are non-significant values of modularity (95% confidence intervals). Only components with at least 10 nodes were analyzed due to the resolution limit in community detection. As the threshold distance increases, the different components of the network connect to one another until the percolation distance is reached where all the nodes in the network belong to the same component. These figures show the spatial network for the range of threshold distances before the percolation point.

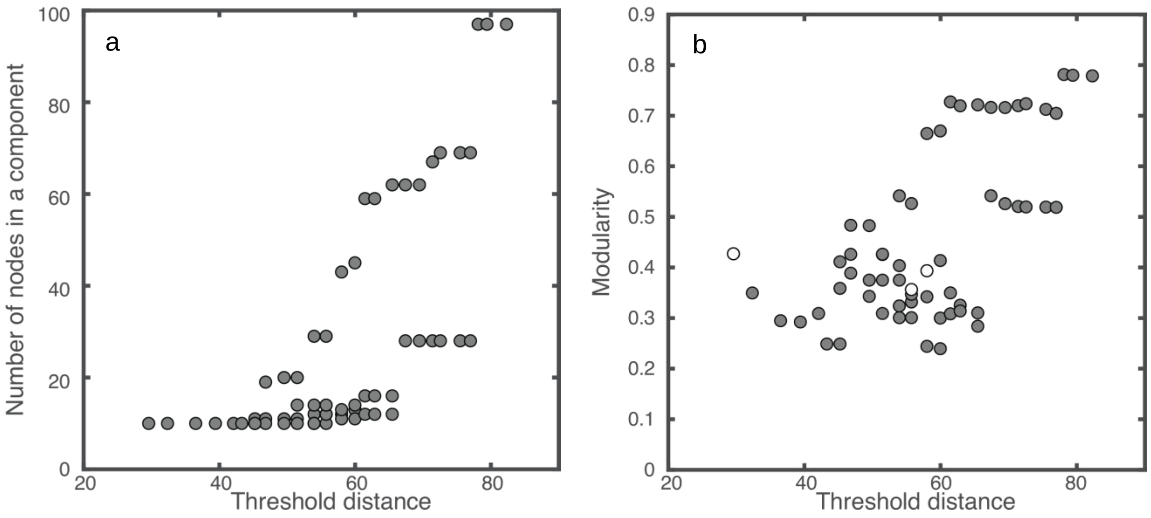

Supplementary Figure 3 | Coordinates from all the peaks above 3000m in the Alps. The main panel shows the modules found in the percolation graph. Value of modularity 0.786, p-val < 0.001. Both histograms show the frequency of nodes across longitude and latitude. The red dotted line represents the average frequency.

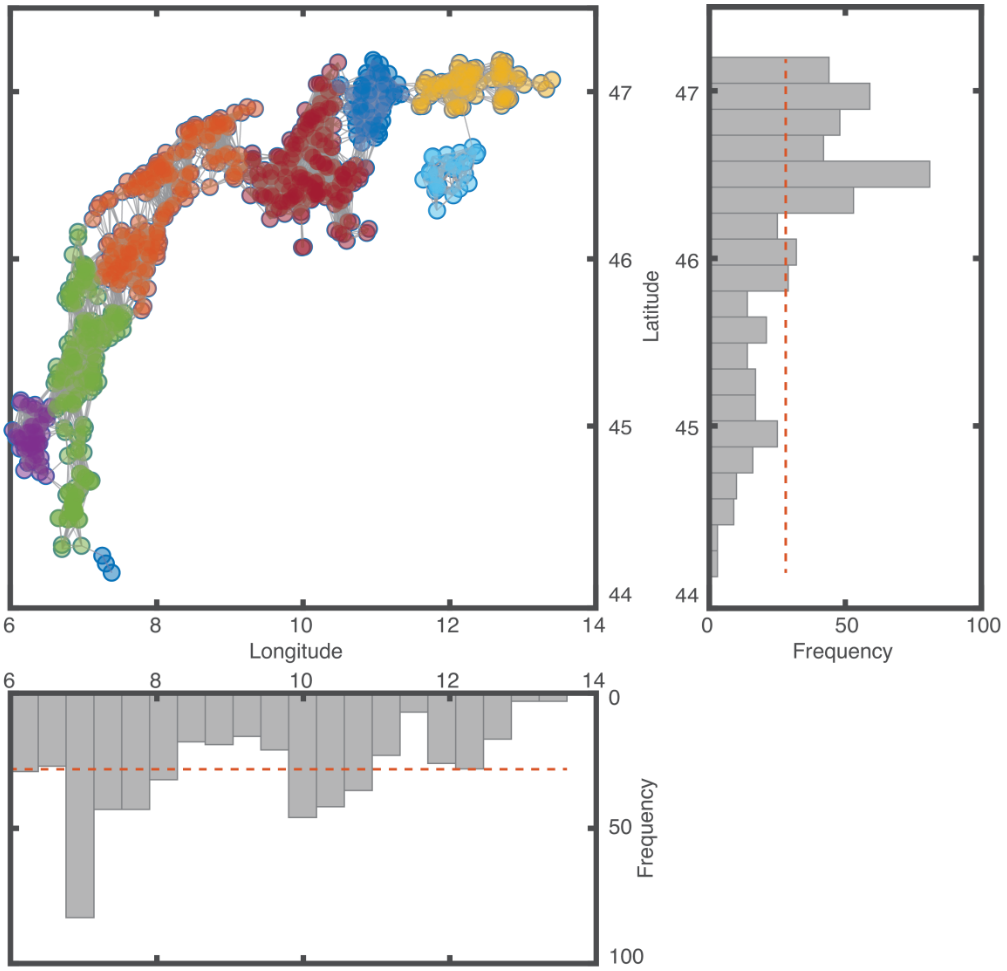

Supplementary Figure 4 | Coordinates from lakes in Yamal, Siberia (Russia). The main panel shows the modules found in the percolation graph. Value of modularity 0.788, p-val < 0.001. Both histograms show the frequency of nodes across longitude and latitude. The red dotted line represents the average frequency.

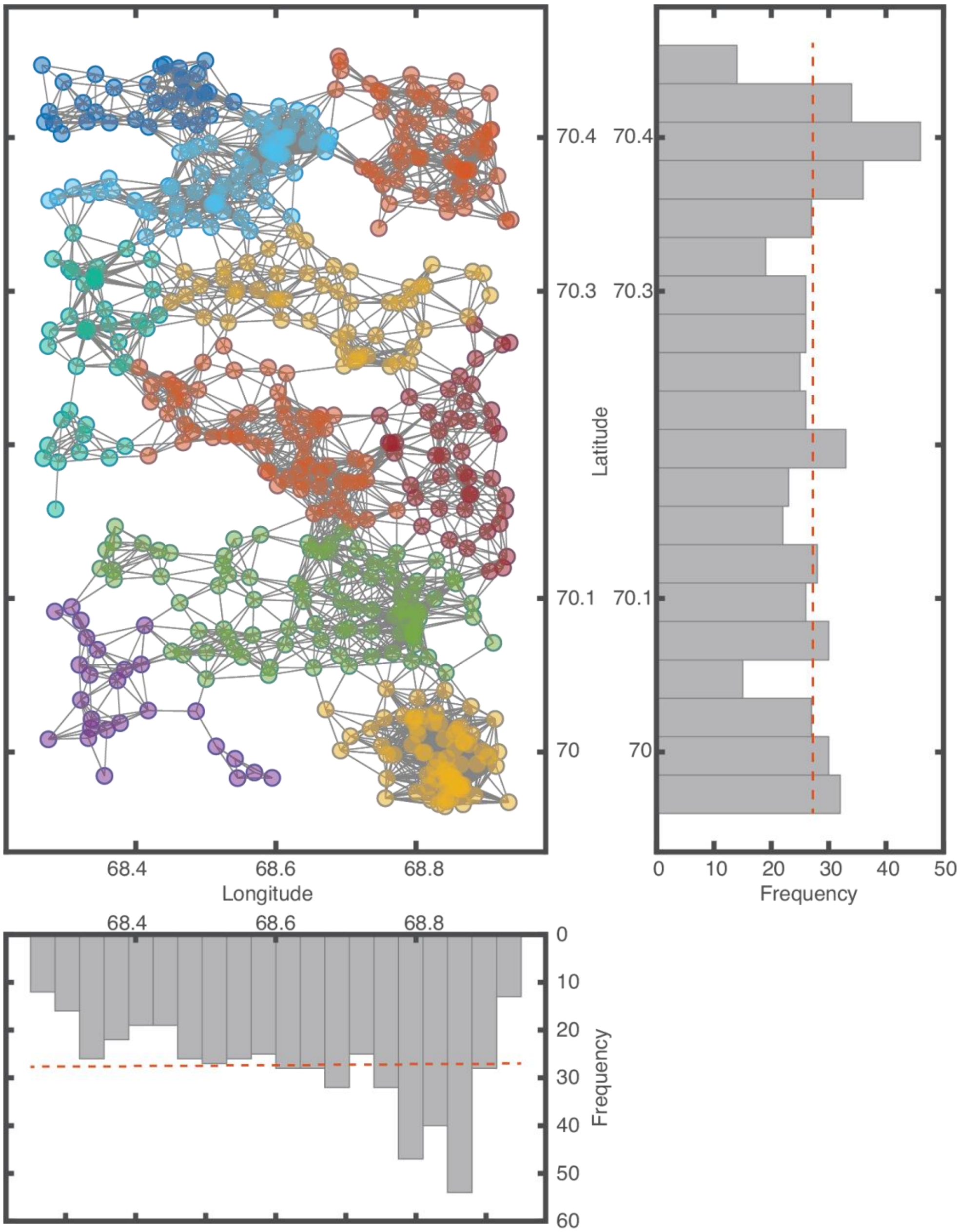

Supplementary Figure 5 | Drawing the coordinates from a normal distribution. This scenario simulates the case in which habitat fragments have a higher density around some coordinates in space, and then the probability of finding a habitat fragment diminishes as the distance from that "hot spot" increases. **a)** Equivalent to Fig S1. **b)** Equivalent to figure 2b in the main text.

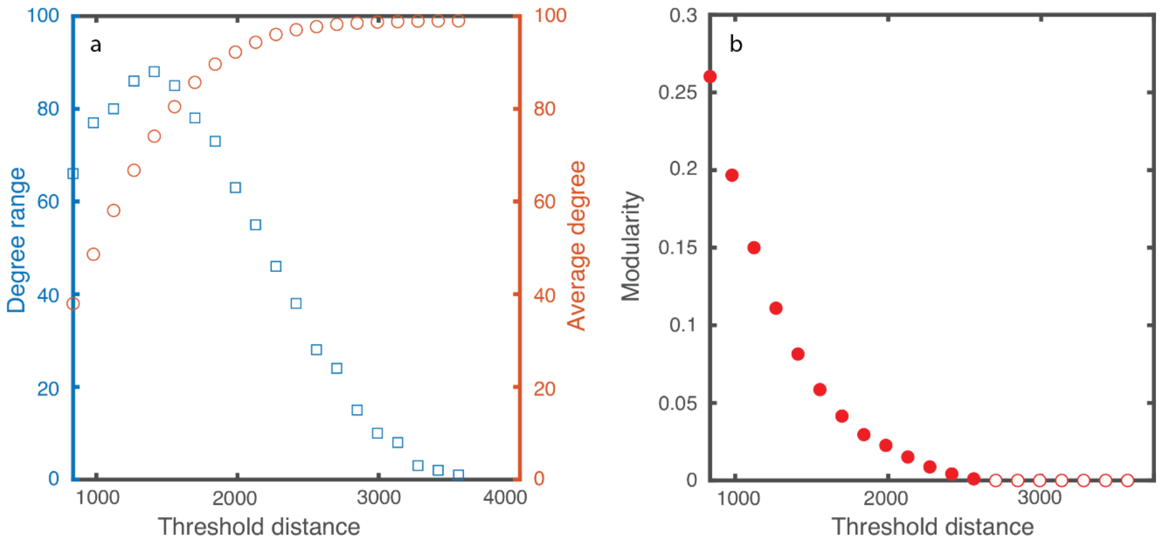

Supplementary Figure 6 | Node density as a function of the distance to a focal node. a) A visual guide on the methodology to calculate node density. Node density around a focal node is calculated as the number that falls within radius "d" divided by the area that falls within the latitude and longitude limits of the landscape. **b)** Considering all nodes, the standard deviation of node density decreases as the distance to a focal node increases.

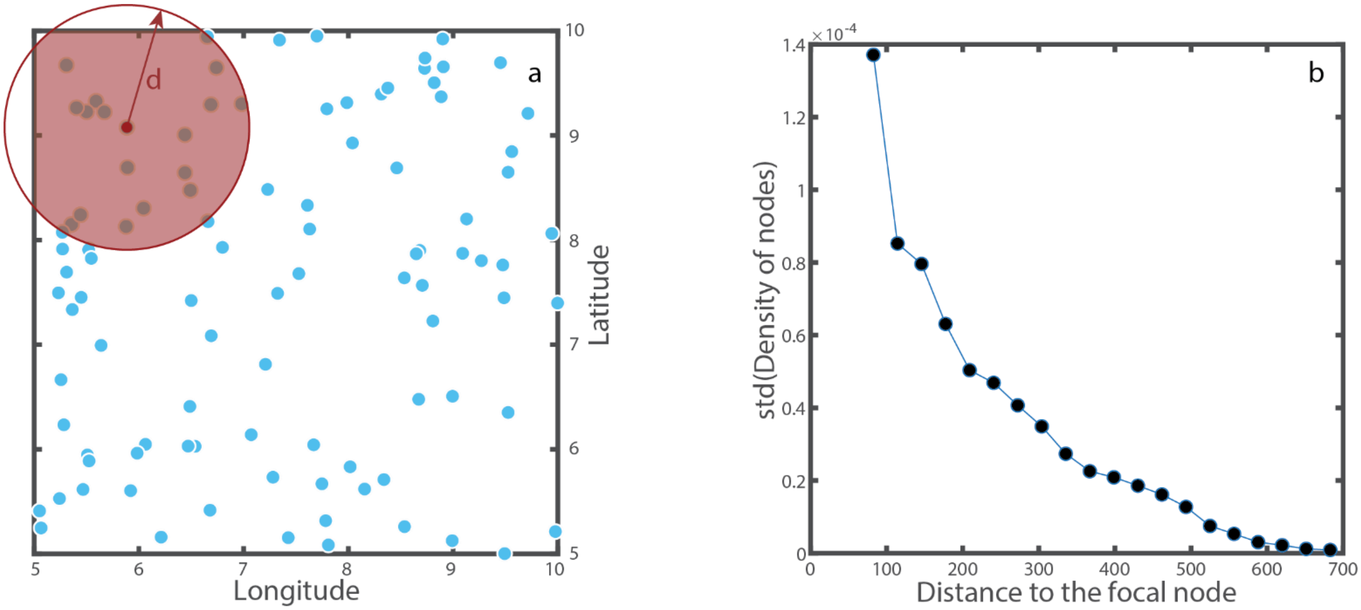

Supplementary Figure 7 | Modularity and node density in the Alps and Yamal peninsula empirical examples. a-c, Binary network modularity as a function of threshold distance for the Alps (**a**) and Yamal peninsula (**c**). Red dotted line represents the percolation distance. Black dots represent significant values of modularity. **b-d**, Considering all nodes, the standard deviation of node density decreases as the distance to a focal node (threshold distance) increases, for the Alps (**b**), and for Yamal peninsula (**d**).

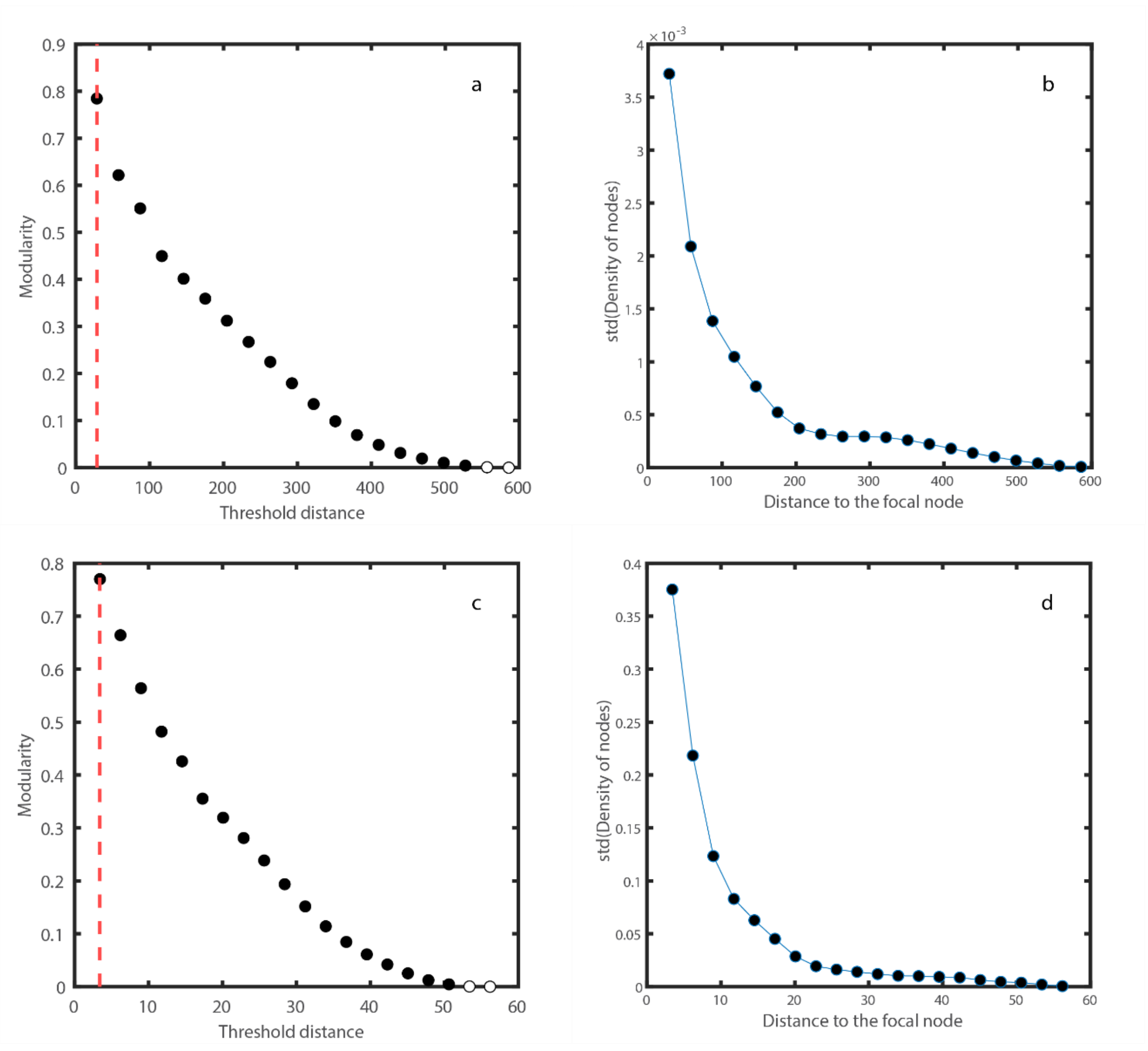

Supplement: Supplementary file 1 — Supplementary Information. [file 41598_2020_65669_MOESM1_ESM.pdf]
